# *ANXA2P2* and *PA2G4P4* Pseudogenes Are Associated with the Response to Ionizing Radiation and Could Be Used as Potential Biomarkers: In Silico Study

**DOI:** 10.3390/biomedicines14010200

**Published:** 2026-01-16

**Authors:** Tomasz Kolenda, Piotr Białas, Kacper Kamiński, Maria Dziuba, Małgorzata Czernecka, Aleksandra Leszczyńska, Kacper Guglas, Joanna Kozłowska-Masłoń, Paulina Poter, Klaudia Dudek, Nina Grzejda, Karina Tylkowska, Anna Zapłata, Marlena Janiczek-Polewska, Paulina Gieremek, Katarzyna Regulska, Patrycja Mantaj, Anna Florczak-Substyk, Anna Przybyła, Urszula Kazimierczak, Ewa Leporowska, Zefiryn Cybulski, Beata Stanisz, Anna Teresiak

**Affiliations:** 1 Research and Implementation Unit, Greater Poland Cancer Center, Garbary Street 15, 61-866 Poznan, Poland; katarzyna.regulska@wco.pl (K.R.); zefiryn.cybulski@wco.pl (Z.C.); 2 Microbiology Laboratory, Greater Poland Cancer Centre, Garbary Street 15, 61-866 Poznan, Poland; 3 Department of Cell Biology, Poznan University of Medical Sciences, 5D Rokietnicka Street, 60-806 Poznan, Poland; 4 Department of Cancer Immunology, Chair of Medical Biotechnology, Poznan University of Medical Sciences, 8 Rokietnicka Street, 60-806 Poznan, Poland; k.kaminski97@gmail.com (K.K.); 5 Department of Histology and Embryology, Poznan University of Medical Sciences, 6 Święcickiego Street, 60-781 Poznan, Poland; 6 Laboratory of Cancer Genetics, Greater Poland Cancer Center, Garbary Street 15, 61-866 Poznan, Poland; klaudia.dudek.16.kd@gmail.com (K.D.); 7 Institute of Human Biology and Evolution, Faculty of Biology, Adam Mickiewicz University, Uniwersytetu Poznańskiego 6, 61-614 Poznan, Poland; 8 Department of Tumor Pathology and Prophylactics, Poznan University of Medical Sciences, Garbary Street 15, 61-866 Poznan, Poland; 9 Department of Tumor Pathology, Greater Poland Cancer Center, Garbary Street 15, 61-866 Poznan, Poland; 10 Faculty of Food Science and Nutrition, Poznan University of Life Sciences, Wojska Polskiego 28, 60-637 Poznan, Poland; 11 Faculty of Biology, Adam Mickiewicz University, Uniwersytetu Poznańskiego 6, 61-614 Poznan, Poland; 12 Department of Laboratory Diagnostics, Greater Poland Cancer Centre, 15 Garbary Street, 61-866 Poznan, Poland; 13 Department of Clinical Oncology, Greater Poland Cancer Centre, 15 Garbary Street, 61-866 Poznan, Poland; 14 Department of Electroradiology, Poznan University of Medical Sciences, 61-701 Poznan, Poland; 15 Department of Pharmaceutical Chemistry, Collegium Pharmaceuticum, Poznan University of Medical Sciences, Rokietnicka 3, 61-806 Poznan, Poland; bstanisz@ump.edu.pl (B.S.); 16 Pharmacy, Greater Poland Cancer Centre, Garbary Street 15, 61-866 Poznan, Poland; 17 Radiation Protection Department, Greater Poland Cancer Centre, Garbary Street 15, 61-866 Poznan, Poland; 18 Department of Diagnostics and Cancer Immunology, Greater Poland Cancer Centre, Garbary Street 15, 61-866 Poznan, Poland

**Keywords:** HNSCC, pseudogene, biomarker, oncogene, TCGA, HPV

## Abstract

**Background:** Head and neck squamous cell carcinoma remains a highly aggressive malignancy with limited predictive biomarkers for prognosis and radiotherapy response. Increasing evidence indicates that pseudogenes are functionally active regulators of cancer biology, yet their clinical relevance in HNSCC is poorly defined. **Methods:** Using transcriptomic and clinical data from The Cancer Genome Atlas, we analyzed the expression and clinical significance of two pseudogenes, *ANXA2P2* and *PA2G4P4*, in HNSCC. Associations with clinicopathological features, HPV status, tumor subtypes, survival, genomic instability, radiotherapy response, and immune landscape were assessed using bioinformatic tools. **Results:** Both pseudogenes were significantly upregulated in HNSCC compared to normal tissues. Higher expression levels correlated with adverse clinicopathological features, increased tumor proliferation and wound-healing capacity, and unfavorable TCGA molecular subtypes. High *ANXA2P2* and *PA2G4P4* expression was associated with reduced overall survival, while their combined low-expression signature identified patients with significantly improved overall and disease-free survival. Notably, lower expression of both pseudogenes was observed in patients responding to radiotherapy, whereas higher expression was linked to genomic instability parameters and enrichment of oncogenic pathways, including MYC, PI3K/AKT/mTOR, cell cycle regulation, and DNA repair. *ANXA2P2* expression differed significantly by HPV status, showing reduced levels in HPV-positive tumors. Furthermore, pseudogene expression stratified distinct immune profiles, including immune subtypes, stromal and immune scores, and specific immune cell populations. **Conclusions:** *ANXA2P2* and *PA2G4P4* are clinically relevant pseudogenes associated with tumor aggressiveness, immune modulation, and radiotherapy response in HNSCC. These findings support their potential utility as prognostic and predictive biomarkers and provide a rationale for further functional validation in experimental models.

## 1. Introduction

Head and neck squamous cell carcinomas (HNSCCs) develop from tissues in the oral cavity, oropharynx, hypopharynx, and larynx. It affects approximately 600,000 new patients yearly. Thus, HNSCCs are the sixth most common type of cancer worldwide [1,2]. The main risk factors include tobacco exposure, overuse of alcoholic beverages, and oncogenic virus infections. Human papillomavirus (HPV) is the most critical viral oncogenic factor contributing to HNSCCs and causes disturbances in the molecular program of cells [3,4,5,6]. Depending on the HNSCC stage, unimodal or multimodal treatment strategies can be applied. In clinical practice, the early stages of HNSCCs are treated by surgery or radiotherapy. In locally advanced cancer, the surgery is followed by adjuvant radiation or chemoradiation [1,2]. It should be noted that despite the progression in chemotherapy, the primary treatment approach for HNSCC is still radiotherapy [7,8,9,10,11,12,13]. In the last decade, much attention has been put on the different types of non-coding RNAs, mostly on microRNAs. Up to date, longer RNA transcripts such as long non-coding RNAs (lncRNAs) also seem essential in HNSCC biology and should be intensively explored as a critical component of the RNA world [14,15,16,17,18].

Pseudogenes are considered copies of genes that have lost their original function due to the accumulation of mutations. The human genome possesses around 11,000 pseudogenes; however, the exact number still remains undefined [19]. Pseudogenes, once considered non-functional “junk DNA,” are now recognized as important genomic elements with potential regulatory functions. Advances in sequencing have revealed their involvement in various biological processes, including their alterations in multiple cancers [18]. Pseudogenes can interact with their parental genes or other genomic loci, influencing their sequence integrity and transcriptional activity. Additionally, pseudogene-derived RNAs can participate in post-transcriptional regulation by acting as antisense RNAs, endogenous small-interfering RNAs, competitive endogenous RNAs (ceRNAs), or through other RNA-mediated mechanisms [19,20,21]. Some pseudogenes may even produce functional proteins that mimic or disrupt the actions of their protein-coding counterparts [19]. The role of most pseudogenes in the pathomechanism of cancer development, including HNSCC, remains poorly understood. Despite the small number of reports about pseudogenes, this type of RNA transcript seems to be a new potential class of biomarkers in modern oncology. For instance, the expression levels of pseudogenes such as *FKBP9P1*, *PTTG3P*, *FTH1P3*, *DUXAP8*, *DUXAP10*, or *PTENP1* were changed during HNSCC progression and could serve as potential diagnostic or prognostic biomarkers [22,23,24,25,26,27,28,29]. In this study, the role of two pseudogenes named *ANXA2P2* (annexin A2 pseudogene 2) and *PA2G4P4* (proliferation-associated 2G4 pseudogene 4) in the pathogenesis of HNSCC and the potential diagnostic utility were analyzed based on the transcriptome data and clinical data obtained during The Cancer Genome Atlas (TCGA) project.

To date, no protein or peptide products have been confirmed for these pseudogenes. *ANXA2P2* has a single transcript (NR_003573.1, 1310 nt), while *PA2G4P4* also has a single transcript (NR_003284.1, 2751 nt), according to the NCBI Gene and Ensembl databases [30,31]. However, the GeneCards database reports an annexin A2-like protein (A6NMY6, 339 amino acids) encoded by *ANXA2P2*, as detected by Komalasari et al. [32,33]. The parental gene of *ANXA2P2*, *ANXA2* (annexin A2), plays a crucial role in cancer progression, including HNSCC. *ANXA2* regulates membrane dynamics, and its downregulation correlates with poorly differentiated and dysplastic tissues in the head and neck region. It is implicated in tumor progression, migration, and metastasis [34,35]. Despite its biological significance, *ANXA2* alone has not been validated as a reliable biomarker for HNSCC monitoring [36,37]. However, a saliva-based protein panel consisting of antibodies against MMP1, KNG1, ANXA2, and HSPA5 demonstrated high sensitivity and specificity in detecting oral squamous cell carcinoma (OSCC) in a Taiwanese population [38]. Furthermore, Quabius et al. reported increased *ANXA2* expression in HPV-DNA-positive tonsillar samples [39]. Notably, *ANXA2* plays a key role in the life cycle of both RNA and DNA viruses, including HPV, hepatitis B (HBV), and hepatitis C (HCV) [40]. The second pseudogene, *PA2G4P4*, originates from *PA2G4* (proliferation-associated 2G4), though little is known about its role in HNSCC. *PA2G4* encodes two protein isoforms: *PA2G4-P42* (which inhibits tumor growth) and *PA2G4-P48* (which promotes tumor growth). Sun et al. demonstrated that *PA2G4-P48* enhances HNSCC proliferation by reducing polyubiquitination via MCTS1 (MCTS1 re-initiation and release factor) [41]. Additionally, cDNA array and proteomic analyses suggest that *PA2G4* mRNA and protein are overexpressed in radioresistant OSCC cell lines; however, Western blot validation yielded inconsistent results [42].

Beyond its role in cancer, *PA2G4* (also known as EBP1, ErbB-3 receptor binding protein) is essential for viral replication. Inhibition of EBP1 induces cell cycle arrest and apoptosis in HIV-infected cells [43]. Similarly, in influenza virus infection, EBP1 expression correlates with viral protein production [44].

Given these findings, we selected *ANXA2P2* and *PA2G4P4* for further investigation in HNSCC. This study aims to explain their biological roles and assess their potential as diagnostic biomarkers in HNSCC using The Cancer Genome Atlas project’ transcriptomic and clinical data.

## 2. Materials and Methods

**Figure 1:**
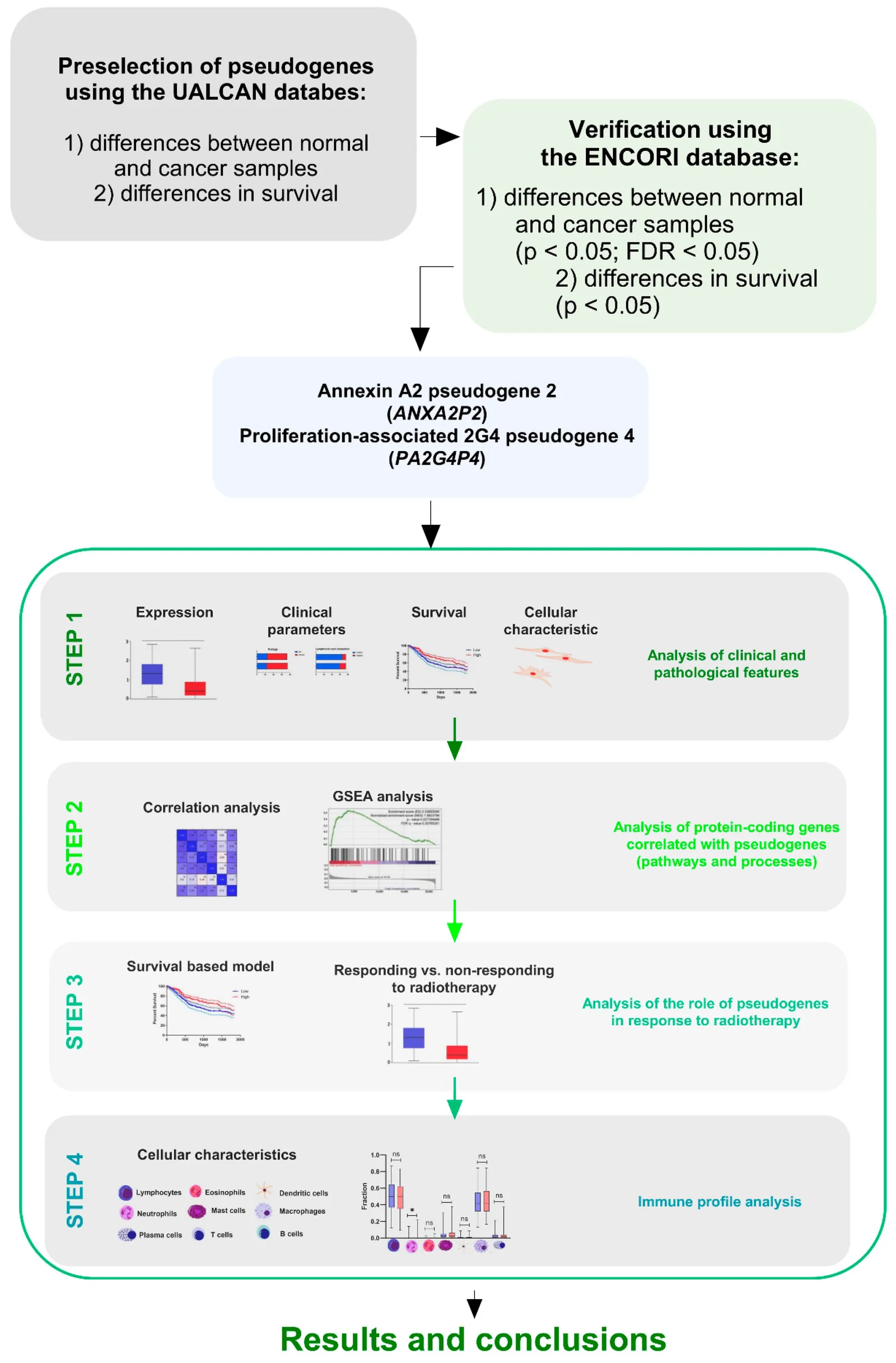
The main steps of the methodology presented in this study based on the preselection using UALCAN and ENCORI databases two pseudogenes, named *ANXA2P2* and *PA2G4P4*, were chosen. Next, using TCGA data downloaded from Santa Cruz UCSC Xena browser analysis was performed including analysis of clinical and pathological features, analysis of protein-coding genes correlated with pseudogenes was performed for description of pathways and processes, analysis of the role of pseudogenes in response to radiotherapy, and immune profile analysis; FDR—false discovery rate; p—probability value; UALCAN—The University of ALabama at Birmingham CANcer data analysis Portal; ENCORI—Encyclopedia of RNA Interactomes; TCGA—The Cancer Genome Atlas; GSEA—Gene Set Enrichment Analysis.

In this study based on the preselection using The University of ALabama at Birmingham CANcer data analysis Portal (UALCAN) database two pseudogenes, named ANXA2P2 and PA2G4P4, were chosen. Next, we checked the data against the Encyclopedia of RNA Interactomes (ENCORI) database [45], using the following criteria; the first: the difference between healthy and cancer tissue with *p* < 0.05 and false discovery rate, FDR < 0.05 and/or the second criteria: difference in patient survival, where *p* < 0.05. Finally, using TCGA data downloaded from Santa Cruz UCSC Xena browser analysis was performed to describe their potential as biomarkers. The main steps of methods are presented in Figure 1.

### 2.1. TCGA Data

The TCGA expression levels data of *ANXA2P2* and *PA2G4P4* genes were downloaded from Santa Cruz UCSC Xena browser (TCGA Head and Neck Squamous Cell Carcinoma, IlluminaHiSeq pancan normalized; 566 samples; UCSC Xena; accession on 20 September 2024) and from UALCAN and ENCORI databases (http://ualcan.path.uab.edu; https://rnasysu.com/encori/; accessed on 20 September 2024) [45,46,47,48].

### 2.2. Pathological and Clinical Analyses

The expression levels of pseudogenes were analyzed in cancerous and corresponding healthy (normal) tissues from the larynx, pharynx, and oral cavity. The HPV status was assessed based on p16 protein (encoded by tumor suppressor gene *CDKN2A*, cyclin-dependent kinase inhibitor 2A) expression and in situ hybridization (ISH) and categorized as positive and negative samples [48], was assessed for all genes using the Wilson/Brown method with a 95% confidence interval (CI). Additionally, correlations between the expression levels of six analyzed pseudogene transcripts were evaluated.

Using the TCGA classification of tumors (atypical, basal, classical, and mesenchymal subtypes), we further analyzed the expression levels of *PA2G4P4* and *ANXA2P2*. Associations between pseudogene expression levels and tumor characteristics, such as proliferation ratio, wound healing capacity, and intratumor heterogeneity, were also determined using data from Thorsson et al. [49]. Furthermore, the expression levels of two pseudogenes were correlated with clinical and pathological parameters; methodologies described by us previously [23,29,45,46,50] and using available online tools like GEPIA2 (Gene Expression Profiling Interactive Analysis, version 2; http://gepia2.cancer-pku.cn/#index; accessed on 18 January 2025) [51].

### 2.3. Gene Analyses and Functional Enrichment Analysis (GSEA)

Genes positively or negatively correlated with pseudogene expression (Spearman’s correlation coefficients R > 0.3 or R < −0.3) were identified using cBioPortal, (https://www.cbioportal.org/; accessed on 20 September 2024). These genes were subsequently analyzed using the REACTOME Pathway Browser (https://reactome.org/; accessed on 20 September 2024), applying a significance threshold of *p* ≤ 0.05. Gene Set Enrichment Analysis (GSEA) was performed using version 4.1.0 of the GSEA software [52], following protocols outlined in prior studies [23,29,45,46,50].

### 2.4. Association of Pseudogenes and Response to Ionizing Radiation

The potential association of *ANXA2P2* and *PA2G4P4* with genomic instability was investigated. Additionally, the expression levels of these pseudogenes were analyzed in the context of HNSCC patients’ responses to radiotherapy, using a model previously described by Paszkowska et al. [29].

### 2.5. Statistical Analyses

All statistical analyses were performed using GraphPad Prism 8 (GraphPad Software, San Diego, CA, USA). Normality was tested using the Shapiro–Wilk test, while comparisons of *ANXA2P2* and *PA2G4P4* expression levels across clinical subgroups were made using *t*-tests or Mann–Whitney U tests, depending on data distribution. Methodologies referenced in previous studies were used where appropriate [23,29,45,46,50].

## 3. Results

### 3.1. Expression of Pseudogenes Is Upregulated in HNSCC and Correlated with Tumor Localization, Tumor Type and HPV Status

Based on the UALCAN database, the pseudogenes ANXA2P2 and PA2G4P4 were significantly overexpressed in primary HNSCC samples compared to normal tissues (*p* = 0.01172 and *p* < 0.0001, respectively) and patients with low/medium expression levels of these pseudogenes displayed better survival than those with higher expression levels (*p* = 0.048 and *p* = 0.037, respectively), Supplementary Figure S1A. Moreover, we verified those data using a different database named ENCORI. We observed that no differences were identified for ANXA2P2 (ENSG00000231991) pseudogene, the fold change FC = 1.10; *p* = 0.17 with FDR = 0.3; but in the case of PA2G4P4 (ENSG00000230457), FC = 1.68; *p* < 0.0001 with FDR < 0.0001. Next, the association between analyzed pseudogenes and patients’ survival was verified using the LongRank test with estimation of coefficient β (Coef β) and hazard ratio (HR). We indicated that in the case of ANXA2P2 pseudogene, we found that patients with lower expression levels of ANXA2P2 had better overall survival (OS) than those with higher expression of this gene (Coef β = 0.41; HR = 1.5; *p* = 0.0032). However, for PA2G4P4, no differences in OS were observed for (Coef β = 0.08; HR = 1.08; *p* = 0.58), Supplementary Figure S1B.

**Figure 2:**
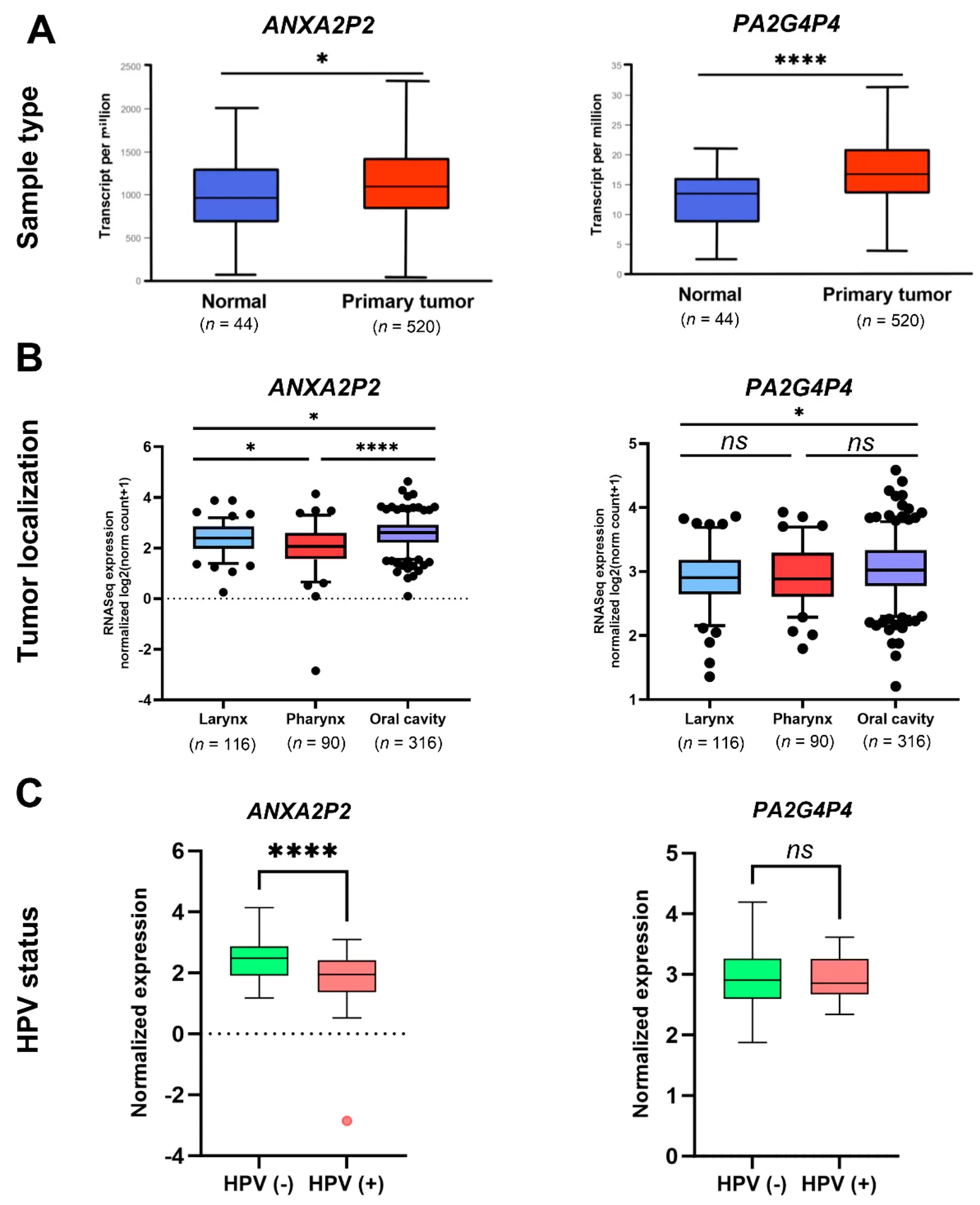
The expression levels of *ANXA2P2* and *PA2G4P4* in HNSCC patients: (**A**) primary tumor and normal tissue samples, un-paired *t*-test; (**B**) Expression levels of all pseudogenes in various HNSCC localizations; mean ranks comparison; box and whiskers with 5–95 percentile, Kruskall-Wallis test obtained using Dunn’s multiple comparisons tests; (**C**) expression between HPV positive and negative groups of patients; ns—not significant, **** *p* < 0.0001, and * *p* < 0.05 considered significant.

Additionally, a strong positive correlation in expression levels was observed between *ANXA2P2* and *PA2G4P4* (Figure 2A). Moreover, we observed that expressions levels of *ANXA2* and *PA2G4* were significantly differ between normal and cancer samples, and in the both cases were upregulated in cancer than in normal type of analyzed samples (*p* = 0.0004 and *p* < 0.00001, respectively), Supplementary Figure S2A. Next, we checked the correlation of parental gene and its pseudogene, *ANXA2* and *ANXA2P2*, *PA2G4* and *PA2G4P4* as well as between two parental genes, *ANXA2* and *PA2G4*, in normal and cancer samples using the GEPIA2 database. We observed, that in the case of *ANXA2* and *ANXA2P2* the higher correlation normal than cancer samples (*p* = 0; R = 0.94, and *p* < 0.0001; R = 0.85, respectively), in the case of *PA2G4* and *PA2G4P4* (*p* < 0.0001; R = 0.56, and *p* < 0.0001; R = 0.33, respectively) as well as *ANXA2* and *PA2G4* (*p* < 0.0001; R = 0.64, and *p* < 0.0001; R = 0.27, respectively). It should be noted that correlation was weaker in cancer samples than in normal in all analyzed by us cases, Supplementary Figure S2B.

To investigate the expression patterns of these pseudogenes based on tumor localization, HNSCC patients were divided into three groups corresponding to the larynx, pharynx, and oral cavity, in accordance with the classification provided by the National Institutes of Health. The analysis revealed the most significant difference in pseudogene expression between tumors from the pharynx and oral cavity (*p* < 0.001), except in the case of *PA2G4P4*, where no differences were observed. Moreover, significant differences in expression levels were also noted for all pseudogenes between tumor samples from the larynx and oral cavity (*p* < 0.05). Among all pseudogenes analyzed, only *ANXA2P2* showed statistically significant differences in expression levels across all tumor localizations (Figure 2B).

Next, the expression levels of the pseudogenes were assessed in relation to the HPV status. The analysis showed that *ANXA2P2* expression was significantly downregulated in HPV-positive patients compared to HPV-negative patients (*p* < 0.0001). However, no significant differences were observed in the expression levels of *PA2G4P4*. Moreover, *ANXA2P2* demonstrated the ability to discriminate between HPV-positive and HPV-negative samples, as indicated by an area under the curve value of 0.7417 (*p* < 0.0001). Additionally, we investigated the expression levels of parental genes depending on the HPV status. Significant upregulation of *ANXA2* expression levels in HPV(−) than the HPV(+) group of patients was observed (*p* = 0.0043) only. In the case of *PA2G4*, no differences were indicated (*p* = 0.336); (Supplementary Figure S2C).

**Figure 3:**
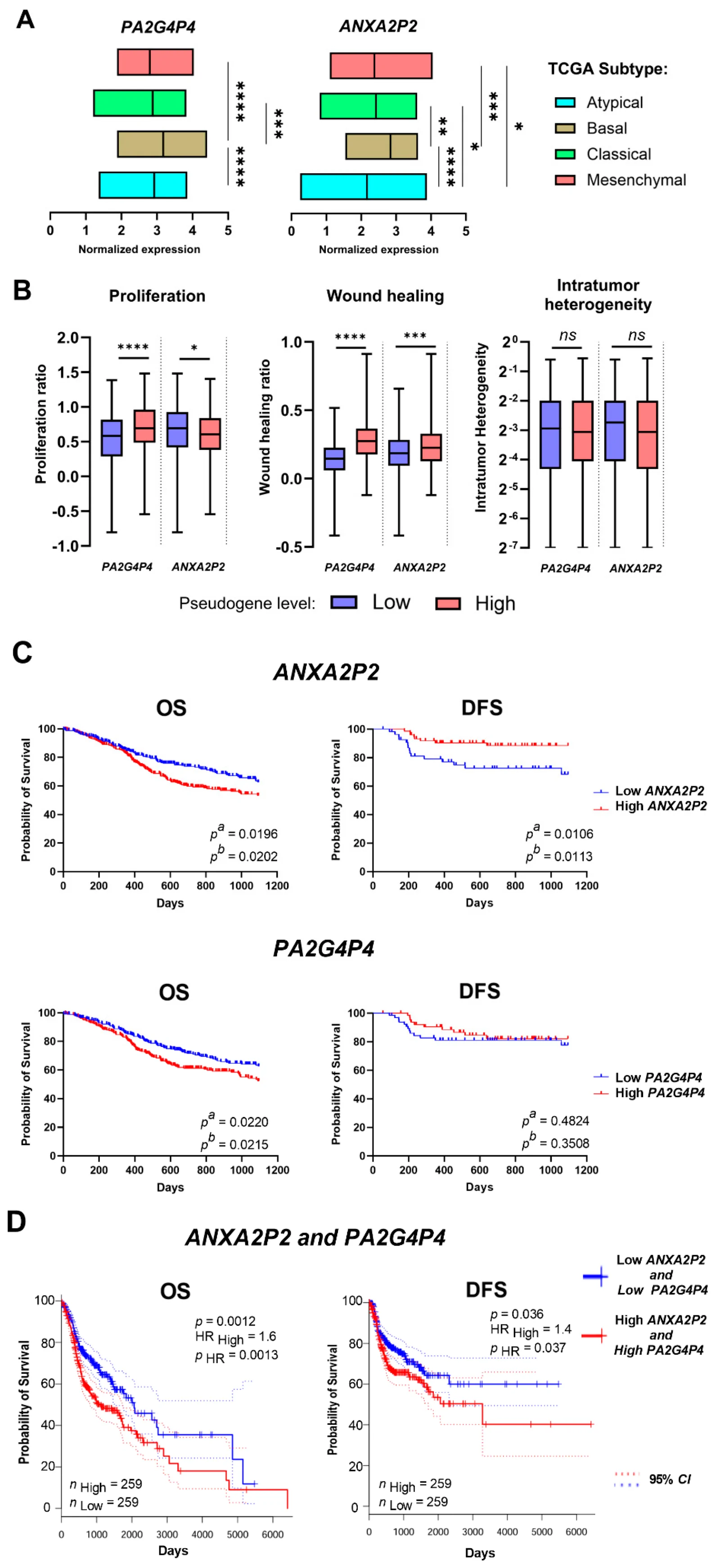
Characteristics of HNSCC tumors depending on *ANXA2P2* and *PA2G4P4* expression levels: (**A**) classification to the atypical, basal, classical and mesenchymal tumor subtype based on the TCGA characteristics; one way ANOVA with Tukey’s multiple comparisons test or Kruskal–Wallis with Dunn’s multiple comparisons test; and (**B**) depending on proliferation ratio, wound healing ability and intratumor heterogeneity; *t*-test or Mann–Whitney U test; ns—not significant, **** *p* < 0.0001, *** *p* < 0.001, ** *p* < 0.01 and * *p* < 0.05 considered significant. (**C**) OS and DFS of HNSCC patients with all localizations of tumors depending on single expression levels of *ANXA2P2* and *PA2G4P4* (results presented for 3 years of observation; *p*^a^—Log-rank (Mantel–Cox) test, *p^b^*—Gehan–Breslow–Wilcoxon test) and (**D**) calculated for signatures expression of two pseudogenes using GEPIA2 tool. Low and high subgroups of patients divided based on mean expression; *p* < 0.05 considered significant; HR—hazard ratio; 95% CI—confidence interval.

Using the TCGA classification of tumors into atypical, basal, classical, and mesenchymal subtypes, the expression levels of *ANXA2P2* and *PA2G4P4* were analyzed further. The findings revealed that atypical subtypes exhibited significantly lower expression levels of both pseudogenes compared to basal subtypes (*p* < 0.0001 for both). In addition, significant differences in expression were noted between basal and classical subtypes for both pseudogenes, (*p* = 0.0003 for *PA2G4P4* and *p* = 0.0092 for *ANXA2P2*). Lower expression levels of *ANXA2P2* and *PA2G4P4* were also observed in mesenchymal subtypes compared to basal subtypes (*p* < 0.0001 and *p* = 0.0001, respectively). For *ANXA2P2*, a significant difference in expression was also detected between atypical and mesenchymal subtypes of HNSCC (*p* = 0.0409); (Figure 3A).

The association of pseudogene expression levels with tumor proliferation, wound healing ability, and intratumor heterogeneity was also evaluated. The results indicated that higher tumor proliferation ratios were observed in patients with elevated levels of *ANXA2P2* and *PA2G4P4*, with *p* < 0.0001 and *p* = 0.0412, respectively. Similarly, increased wound-healing ability was associated with higher expression levels of *ANXA2P2* and *PA2G4P4* (*p* < 0.0001 and *p* = 0.0007, respectively). In contrast, no significant associations were observed between pseudogene expression levels and intratumor heterogeneity (Figure 3B and Supplementary Table S2).

### 3.2. Expression Levels of Pseudogenes Differ Depending on Clinicopathological Parameters

The expression levels of *ANXA2P2* and *PA2G4P4* were correlated with various clinicopathological parameters of HNSCC patients, including age, gender, smoking status, and cancer stage. Significant differences were observed between pseudogene expression levels and specific parameters. Notably, the expression of *ANXA2P2* showed a significant association with gender (*p* = 0.0079), T-stage (*p* = 0.0427), N-stage (*p* = 0.0314), tumor grade (*p* = 0.0033), and perineural invasion (*p* = 0.0116). For *PA2G4P4*, significant differences were identified in relation to N-stage (*p* = 0.0226). All results are summarized in Table 1.

### 3.3. Patients with Low Expression Levels of ANXA2P2 and PA2G4P4 Display Better Survival Rates

HNSCC samples were divided into low- and high-expressing groups based on the mean level of the studied pseudogene. The differences in disease-free survival (DFS) and overall survival (OS) between these patient groups were calculated. Significantly longer OS was observed in the group of patients with low *ANXA2P2* and *PA2G4P4* expression (*p* = 0.0202, *p* = 0.0215, respectively) compared to patients characterized by higher expression, Figure 3C. The difference in DFS was discovered in patients with low *ANXA2P2* expression levels compared to high-expressing patients. However, the OS calculated for signatures expression of two pseudogenes using the GEPIA2 tool indicated that patients with the jointly lower expression level of *ANXA2P2* and *PA2G4P4* had significantly longer survival time (*p* = 0.0012) and present better DFS (*p* = 0.037); (Figure 3D). Furthermore, the OS and DFS analysis for parental genes of *ANXA2P2* and *PA2G4P4* showed that patients presented no differences in DFS (*p* = 0.14) and only slightly better OS for the group with lower expression levels in comparison to patients with higher levels of both of those protein-coding genes (*p* = 0.048); Supplementary Figure S2D.

### 3.4. Pseudogene Expression Is Associated with Several Cellular Processes and Pathways

**Figure 4:**
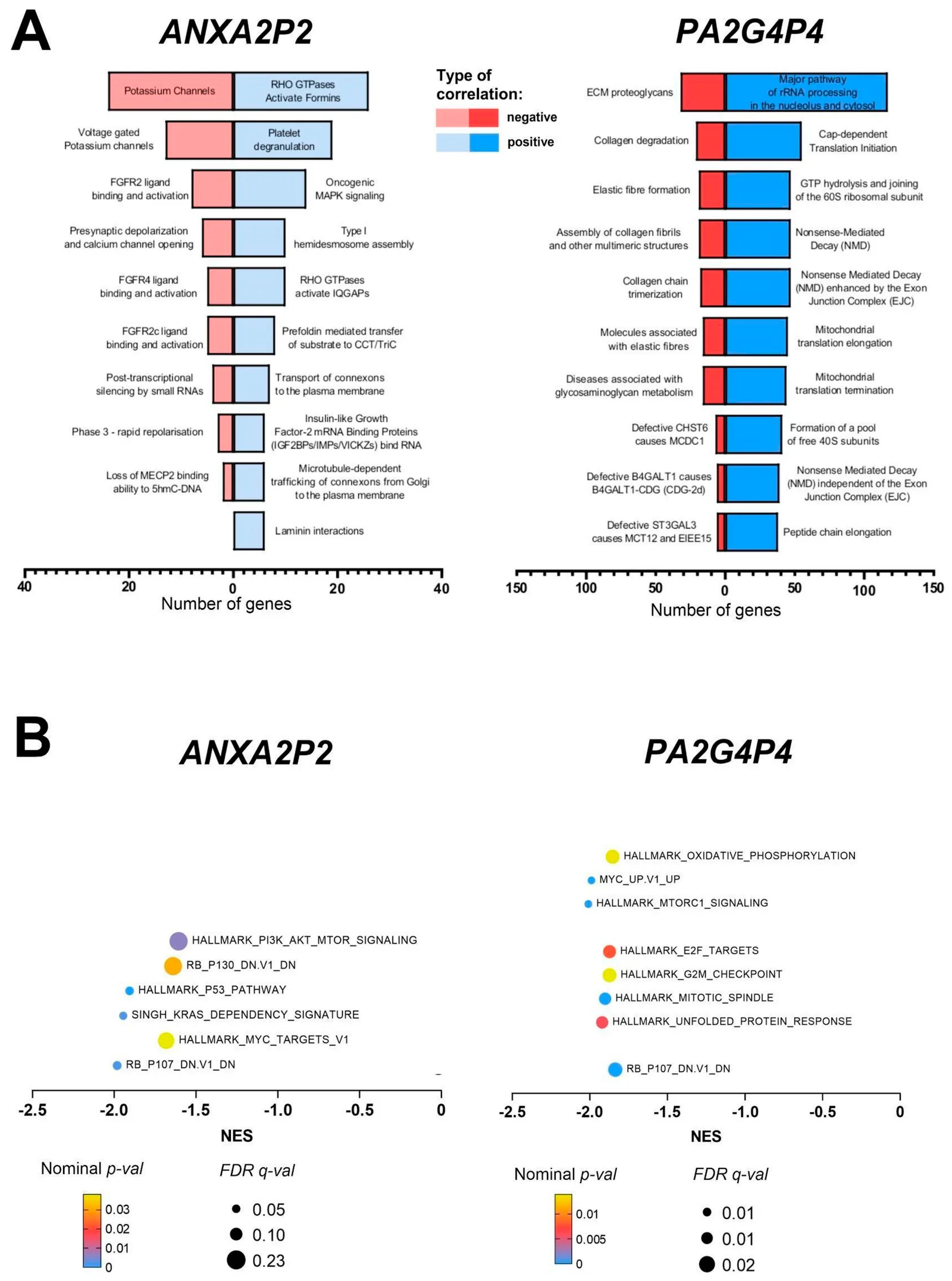
(**A**) Classification of negative (red color) and positive (blue color) correlation of *ANXA2P2* and *PA2G4P4* with genes involved in important cellular processes and pathways. Only genes with Spearman’s correlation >0.3, <−0.3, and *p*-value < 0.05 were included in REACTOME pathway analysis. Darker red and blue colors indicate *FDR* < 0.05 and lighter colors indicate *FDR* > 0.05. (**B**) Significantly enriched genes implicated in the signature gene hallmark and oncogenic based on GSEA between groups of patients with low and high expression of *ANXA2P2* and *PA2G4P4* pseudogenes. *NES*—normalized enrichment score, count—number of genes that are enriched in a specified deregulated process; only results set with *p* ≤ 0.05 and *FDR* ≤ 0.25 were shown.

Gene sets from cBioPortal with Spearman’s negative correlations (R < −0.3) and positive correlations (R > 0.3) with the analyzed pseudogenes were selected and subjected to pathway analysis using REACTOME to classify them into critical cellular processes and pathways (*p* < 0.05). For *ANXA2P2*, the most negatively correlated genes were involved in regulating potassium channels, opening calcium channels, FGFR2 ligand binding and activation, and genes associated with platelet degranulation. Conversely, the most positively correlated genes were linked to RHO GTPase activation and formin-mediated pathways related to platelet degranulation. For *PA2G4P4*, the negatively correlated genes were primarily involved in collagen degradation, collagen chain trimerization, and diseases related to glycosaminoglycan metabolism (Figure 4A and Supplementary Table S3).

To further explore the relationship between pseudogene expression and patient gene expression profiles, Gene Set Enrichment Analysis (GSEA) was performed. This analysis calculated the enrichment of gene sets in patients with high- and low-expression of the respective pseudogenes. In the case of *ANXA2P2*, significantly enriched gene sets were associated with high pseudogene expression. In contrast, *PA2G4P4* demonstrated more statistically significant enrichment in patients with low pseudogene expression. Patients with high levels of *ANXA2P2* presented enrichment in deregulated genes associated with the p53 signaling pathway, MYC targets, and genes upregulated by activation of the PI3K/AKT/mTOR pathway. Additional oncogenic signatures included dysregulation of genes downregulated in primary keratinocytes and genes defining the KRAS dependency signature. On the other hand, patients with high levels of *PA2G4P4* showed enrichment in genes regulated by MYC, upregulated by the mTORC1 complex or during the unfolded protein response, and associated with DNA repair mechanisms, the G2/M checkpoint, and cell cycle regulation through E2F transcription factors. Furthermore, pathways linked to oxidative phosphorylation were also significantly associated with high *PA2G4P4* expression (Figure 4B and Supplementary Table S4).

### 3.5. ANXA2P2 and PA2G4P4 Pseudogenes Are Associated with the Response to Ionizing Radiation

Analysis of cellular pathways revealed that the studied pseudogenes, *ANXA2P2* and *PA2G4P4*, are associated with critical pathways involved in the cellular response to ionizing radiation. To explore the potential association of these pseudogenes with genome stability and the response to radiotherapy in HNSCC patients, we applied the model previously described by Paszkowska et al. [29].

**Figure 5:**
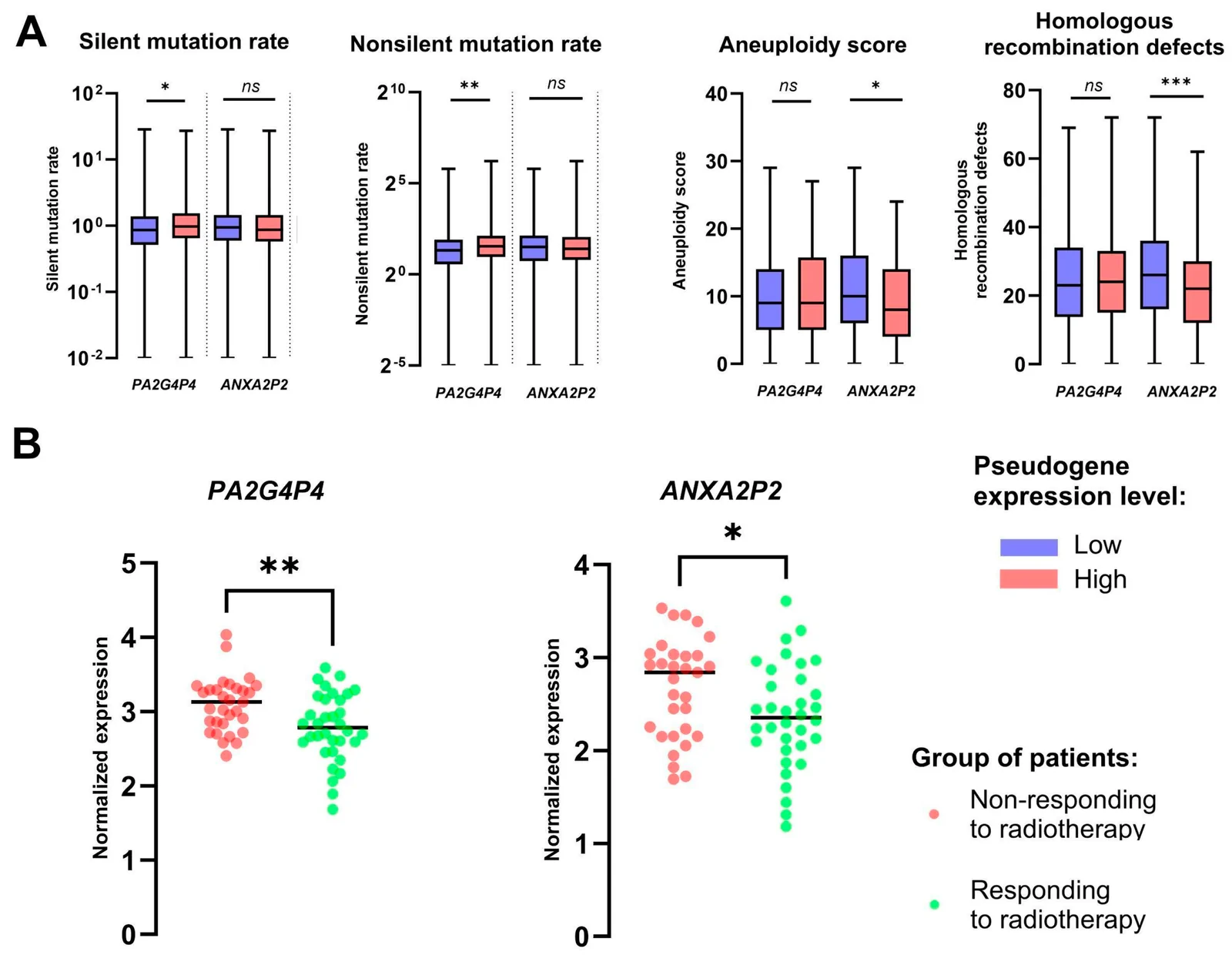
Association of *ANXA2P2* and *PA2G4P4* pseudogenes with genome instability and response to radiotherapy of HNSCC patients: (**A**) silent and nonsilent mutation rates, aneuploidy score and homologous recombination defects in the group of patients with low and high expression levels of specified pseudogenes; (**B**) differences in response to radiotherapy depending on the expression levels of *ANXA2P2* and *PA2G4P4* pseudogenes assessed based on the model described by Paszkowska et al. [29]; *t*-test or Mann–Whitney U test; ns—not significant, *** *p* < 0.001, ** *p* < 0.01 and * *p* < 0.05 considered as significant.

Our findings demonstrated that for *PA2G4P4*, significant differences in silent and nonsilent mutation rates were observed between patients with high- and low-expression levels of this pseudogene (*p* = 0.0112 and *p* = 0.0017, respectively). Furthermore, patients with higher *ANXA2P2* expression levels exhibited significantly lower aneuploidy scores compared to those with lower *ANXA2P2* expression (*p* = 0.0264). Homologous recombination defects, another parameter linked to genome stability, were also studied. It was observed that lower *ANXA2P2* expression was associated with higher homologous recombination defects (*p* = 0.0004). These findings highlight the potential role of *ANXA2P2* and *PA2G4P4* in maintaining genome stability and influencing the response to radiotherapy in HNSCC (Figure 5A and Supplementary Table S5). Finally, based on our previously published model, we check if expression levels of *ANXA2P2* and *PA2G4P4* differ between groups of patients with and without response to radiotherapy. We observed that in the case of both, *ANXA2P2* and *PA2G4P4,* patients with response to radiotherapy have lower expression levels of those pseudogenes in comparison to the group of patients displaying no response to this treatment (*p* = 0.0297 and *p* = 0.0040, respectively); (Figure 5B and Supplementary Table S5).

### 3.6. Immunological Profiles of HNSCC Patient Samples Differ Depending on Pseudogene Levels

**Figure 6:**
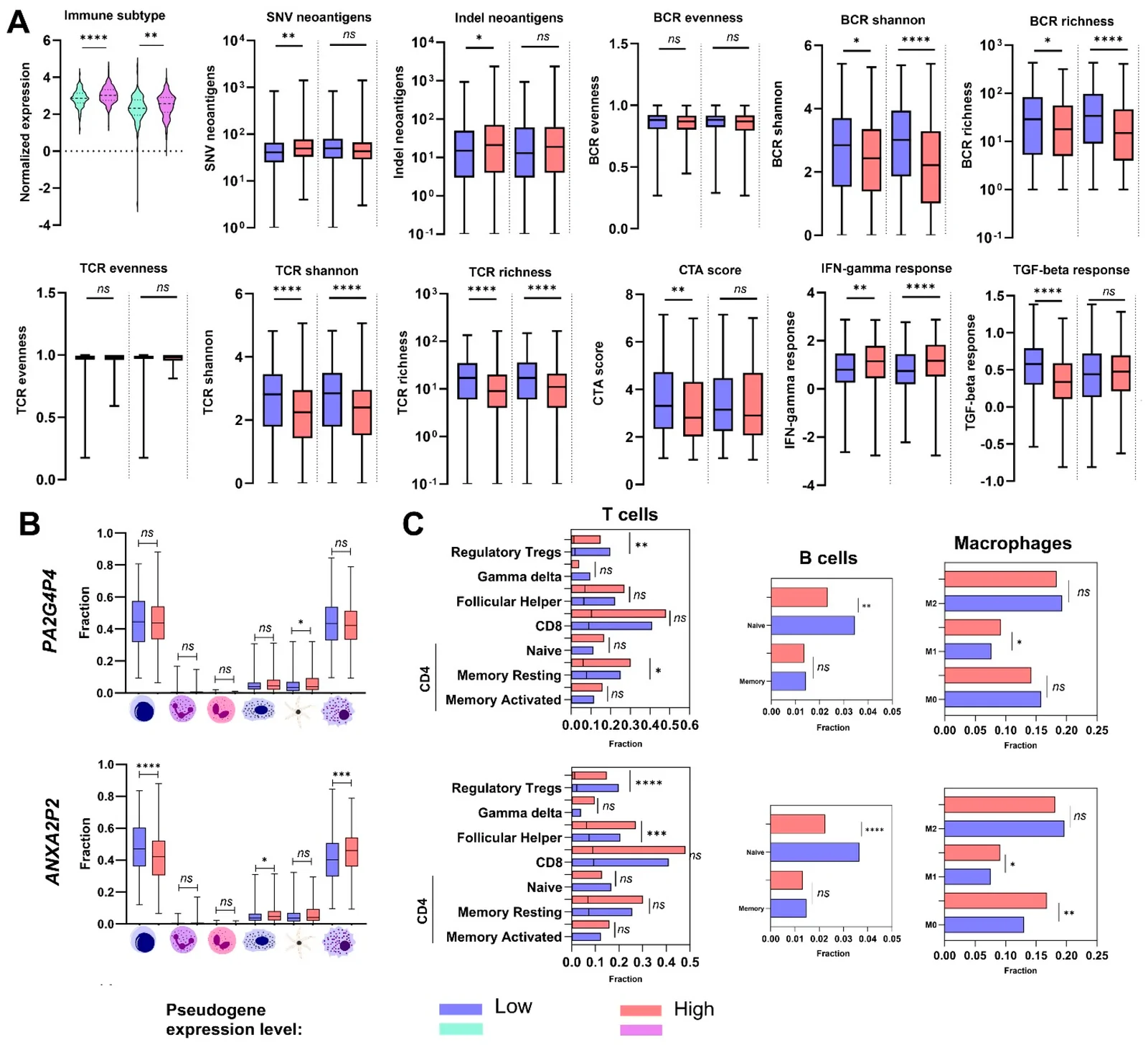
Immunological characteristics of HNSCC patients depending on the expression levels of *ANXA2P2* and *PA2G4P4*: (**A**) TCGA immune subtype (C1 and C2), SNV (single-nucleotide variants) neoantigens, Indel (short insertion and deletion) neoantigens, BCR (B cell receptor) evenness, BCR Shannon, BCR richness, TCR (T cell receptor) evenness, TCR Shannon, TCR richness, CTA (cancer/testis antigen) score, IFN-gamma (interferon gamma) and TGF-beta (transforming growth factor beta) responses in the group of patients with low and high expression levels pseudogenes; (**B**) Differences in the population of lymphocytes, neutrophils, eosinophils, mast cells, dendritic cells and macrophages, (**C**) and fraction of specific subpopulation of T cells, B cells, and macrophages associated with low- and high-expression level of analyzed pseudogenes; *t*-test or Mann–Whitney U test; ns—not significant, **** *p* < 0.0001, *** *p* < 0.001, ** *p* < 0.01 and * *p* < 0.05 considered significant.

Finally, we examined the possible association of the expression levels of *PA2G4P4* and *ANXA2P2* pseudogenes with the immune profile of HNSCC patients. High-expression levels of two pseudogenes were associated with the C2 immune subtype, and low-expression was associated with C1 subtype (*p* < 0.0001 and *p* = 0.0049, respectively). In the case of SNV (single-nucleotide variants) as well as Indel (short insertion and deletion) neoantigens only for patients with higher levels of *PA2G4P4* were associated with higher neoantigens score than patients with lower levels of this pseudogene (*p* = 0.0022 and *p* = 0.0175, respectively). Next, the B cell receptor (BCR) and T cell receptor (TCR) scoring, depending on the pseudogene expression levels, were calculated. No differences (*p >* 0.05) for all pseudogenes were indicated in the case of BCR and TCR evenness scores. In the case of BCR Shannon and BCR richness, higher expression levels of all analyzed pseudogenes were associated with lower scores (*p* = 0.045, and *p* < 0.0001, respectively, and for BCR richness: *p* = 0.0356, and *p* < 0.0001, respectively). In the case of TCR Shannon as well as TCR richness, the same dependency as for BCR was observed (*p* < 0.0001 and < 0.0001, respectively, and for BCR richness: *p* < 0.0001 and *p* < 0.0001, respectively). For the cancer/testis antigen (CTA) score, no association with *ANXA2P2* was observed, but a lower CTA score was indicated for patients with higher levels of *PA2G4P4* (*p* = 0.0029). The last immunological parameters were the interferon-gamma and transforming growth factor beta responses in patients with low- and high-expression levels of *PA2G4P4* and *ANXA2P*. We observed that higher levels of all analyzed pseudogenes were associated with a higher IFN-gamma response (*p* = 0.0044, and *p* < 0.0001, respectively). In contrast, lower expression levels of *PA2G4P4* were connected with better TGF-beta responses (*p* < 0.0001). No differences in *ANXA2P* and TGF-beta responses were noticed (*p >* 0.05). All data is presented in Figure 6A and Supplementary Table S6.

The ESTIMATE stromal and immune scores were analyzed in HNSCC samples divided into low and high pseudogene expression groups. Significantly lower ESTIMATE scores were observed in HNSCC patients with high expression of *ANXA2P2* and *PA2G4P4* compared to low expression groups (*p* = 0.0467, *p* < 0.0001, respectively). The stromal cell infiltration and lower scores were found in patients with higher expression of *ANXA2P2* and *PA2G4P4* (*p* = 0.0407, *p* < 0.0001, respectively). The immune cell infiltration was significantly lower in patients with high expression of *PA2G4P4* (*p* = 0.0003). All results are shown in Supplementary Table S7.

Next, the immunological profiles of HNSCC patients with various expression levels of pseudogenes, based on the immune score analysis were assessed. Accordingly, fractions of lymphocytes, neutrophils, eosinophils, mast cells, dendritic cells, and macrophages were evaluated. A significantly smaller fraction of dendritic cells was observed in HNSCC patients with low *PA2G4P4* expression (*p* = 0.0197); in the case of *ANXA2P2* for patients with lower expression of these pseudogenes, we observed higher levels of lymphocytes (*p* < 0.0001) and lower levels of macrophages (*p* = 0.0031). Moreover, a higher fraction of mast cells (*p* = 0.0112) was found in the group of patients with higher expression of *ANXA2P2* than in the group with lower expression of this pseudogene, Figure 6B,C, and Supplementary Table S7. In the case of the *PA2G4P4*, differences were found in the subpopulation of CD4 memory resting T cells, regulatory Tregs, naive B cells, and macrophages type M1 (*p* = 0.0210, *p* = 0.0019, *p* = 0.0034, *p* = 0.0086, respectively). For patients with higher levels of *ANXA2P2,* a lower fraction of follicular helper, regulatory Tregs, naive B cells, and a higher fraction of M0 as well as M1 macrophages were found (*p* = 0.0004, *p* < 0.0001, *p* < 0.0001, *p* = 0.0011, and *p* = 0.0105, respectively). All results are presented in Figure 6B,C and Supplementary Table S7.

## 4. Discussion

Over the past few decades, substantial efforts have been dedicated to identifying reliable biomarkers for head and neck squamous cell carcinoma, aiming to enhance diagnosis, prognostication, and therapeutic outcomes. These efforts have been particularly focused on enabling personalized approaches to HNSCC management. This includes the development of cellular-, DNA-, and RNA-based markers from diverse sources and the improvement of imaging modalities such as dual-time-point fluorine-18-fluorodeoxyglucose positron emission tomography/computed tomography (DTP 18F-FDG PET/CT). These advancements hold promise for better-predicting disease progression, patient survival, and treatment responses, including radiotherapy [5,6,17,53,54,55,56,57,58].

Its parental gene, ANXA2, is well-studied and implicated in promoting cancer cell proliferation, migration, invasion, metastasis, treatment resistance, and immune modulation in various malignancies. In HNSCC, ANXA2 is upregulated in laryngeal and oral squamous cell carcinoma tissues and fluids and correlates with advanced disease and poor prognosis [38,59,60,61].

In this study, we analyzed the expression of two pseudogene transcripts: ANXA2P2 and PA2G4P4 and found them to be significantly overexpressed in HNSCC tissue samples compared to adjacent normal tissues. Based on these findings, we propose *ANXA2P2* and *PA2G4P4* as potential biomarker candidates for HNSCC. *ANXA2P2* is one of three pseudogenes of annexin A2, located on chromosome 9 (9p13.3), and shares high sequence homology with annexin A2. Its overexpression has been reported in hepatocellular carcinoma, where it is associated with an aggressive phenotype [62]. Higher *ANXA2P2* expression has also been linked to the severity of diffuse gliomas, suggesting its potential as a prognostic biomarker [63]. To our knowledge, this is the first report linking *ANXA2P2* expression to HNSCC. However, its parental gene, *ANXA2*, is well-studied and implicated in promoting cancer cell proliferation, migration, invasion, metastasis, treatment resistance, and immune modulation in various malignancies. In HNSCC, *ANXA2* is upregulated in laryngeal and oral squamous cell carcinoma tissues and fluids and correlates with advanced disease and poor prognosis [38,59,60,61].

*PA2G4P4*, located on the long arm of chromosome 3 (3q25.31), resides within the region of the long intergenic non-coding RNA 886 (LINC00886). Its functional counterpart, *PA2G4*, is located on chromosome 12 and encodes ErbB3-binding protein (EBP1), which plays a pivotal role in many cancers [64,65,66]. *PA2G4P4* is upregulated in invasive bladder cancer cell lines and patient samples, where it contributes to cancer development by regulating cell proliferation, migration, and apoptosis [65]. In glioblastoma, *PA2G4P4* promotes the expression of the *PA2G4* oncogene and facilitates its nuclear translocation, affecting cell viability and apoptosis [64]. Li et al. analyzed TCGA data and identified *PA2G4P4* as a top-ranked pseudogene essential for tumor classification. Furthermore, overexpression of *PA2G4P4* has been observed in several tumor types, including HNSCC, ovarian cancer, and sarcoma [67]. Together, these findings underscore the oncogenic potential of *ANXA2P2* and *PA2G4P4* and justify further investigation into their clinical and mechanistic roles in HNSCC.

Recent studies suggest that pseudogenes are not merely transcriptional noise or non-functional “junk DNA,” but rather play crucial roles in cellular processes. Pseudogene-derived RNAs can act as competitive endogenous RNAs (ceRNAs), antisense RNAs, or serve as sources of small interfering RNAs (siRNAs), thereby influencing the expression of other genes involved in cancer development and progression. Furthermore, pseudogenes may affect the sequence integrity and transcriptional regulation of their parental genes. These regulatory functions may contribute significantly to the initiation and progression of HNSCC, especially through complex post-transcriptional networks and feedback mechanisms that remain to be fully explained.

We also observed a significant association between the expression levels of these pseudogenes and tumor localization, particularly in tumors originating from the pharynx and oral cavity. We hypothesize that the tumor localization of the localization-specific expression may reflect regionally distinct oncogenic functions of these pseudogenes. Additionally, expression levels positively correlated with key clinicopathological parameters such as cancer stage, T-stage, N-stage, and tumor grade. That supports our hypothesis of a potential oncogenic function of the above pseudogenes in HNSCC. However, other mechanisms, such as changes in cellular transcription during cancer progression, cannot be excluded.

Importantly, ANXA2P2 expression differed between HPV(−) and HPV(+) patients. In our previous work, we indicated that *PTTG3P* is associated with HPV infection in HNSCC [23]. Santini et al. showed that *ANXA2P2* is downregulated in invasive cervical carcinomas in comparison to normal cervical keratinocytes. The above supports our observation in the HPV(+) patients displaying lower levels of the transcript [68]. Moreover, the *ANXA2T* (annexin A2 heterotetramer) is involved in HPV endocytosis and capsid disassembly as well as in virion degradation prevention [59]. It should be noted that *ANXA2* is involved in immune response [68], but the role of its pseudogene was not yet validated. We did not observe changes in the immune profile of HNSCC patients associated with the expression level of *ANXA2T.*

We also performed the gene set enrichment analysis to find positive and negative correlations between *ANXA2P2* and *PA2G4P4*, and different biological processes. We found that genes correlated with the analyzed pseudogenes were associated with oncogenic processes. *ANXA2P2* is positively correlated with formins activation by RHO GTPases and oncogenic MAPK signaling pathways. Formins are involved in regulating the actin cytoskeleton and in cancer cell motility and invasiveness [69]. On the other hand, the RAS/RAF/MAPK pathway regulates cellular proliferation, differentiation, and survival, and is frequently mutated in human cancers [70]. GSEA also indicated that patients with high expression of *ANXA2P2* and *PA2G4P4* have dysfunctions in carcinogenesis pathways, which suggests that they are oncogenic pseudogenes. Moreover, we observed that patients with lower expression of analyzed pseudogenes displayed lower enrichment of genes involved in the oncogenic signature gene sets in comparison to high expression groups. The cellular pathways that are often dysregulated in cancer may lead to aggressive cellular phenotype. The patients with lower expression of specific pseudogenes may display profiles like cells with BMI1 knockdown or downregulated genes associated with epithelial-to-mesenchymal transition process or longer survival, indicating the oncogenic function of studied pseudogenes in HNSCC [71].

In the treatment of HNSCC, radiotherapy represents a crucial therapeutic modality and serves as the standard of care in palliative settings [72,73,74]. However, radiotherapy is associated with numerous adverse effects, including psychological distress such as procedural stress and anxiety [75], radiation-induced skin injury and radiation dermatitis in face area [76,77], and radiation induced brachial plexopathy [78], as well as problems with assessment of margin and high uncertainties with decision making between avoiding recurrence and protection of critical organs [79,80,81]. These limitations underscore the need for strategies aimed at optimizing radiotherapy efficacy and enabling biological assessment of the response to ionizing radiation [56,82,83,84,85]. One approach is epigenetic analysis and the search for epigenetic patterns that can serve as predictive biomarkers in the personalization of radiotherapy for head and neck cancers. Previously, we explored the expression of pseudogenes *AURKAPS1*, *HERC2P2*, and *SDHAP1* in the context of radiotherapy response. *AURKAPS1* showed predictive value, with low expression associated with improved response and decreased DNA repair gene expression, suggesting a role in modulating radiosensitivity through genomic stability [46]. Moreover, *AURKAPS1* was associated with changes in DNA repair. Patients with lower levels of *AURKAPS1* had a better response to radiotherapy and displayed lower levels of DNA repair genes. It is probably a direct link between lower expression levels of DNA repair genes and better patients’ response to radiotherapy. And probably *AURKAPS1* has the same biological role in this phenomenon [46].

Here, we extended this line of inquiry to *ANXA2P2* and *PA2G4P4* to explore their potential roles in genomic instability and radiotherapy response in HNSCC patients. Limited evidence exists regarding the interplay between pseudogene expression, radiation response, and genomic instability [86]. Our research is the first to describe the *ANXA2P2* and *PA2G4P4* pseudogenes in this context. We observed that patients with higher levels of *ANXA2P2* and *PA2G4P4* responded worse to the radiotherapy than those with lower levels of this pseudogene. Furthermore, pathway enrichment analysis revealed that patients with high *ANXA2P2* expression displayed upregulation of gene sets associated with rb_p107_dn.v1_dn, singh_kras_dependency_signature, p53 pathway, myc targets v1, rb_p130_dn.v1_dn, tbk1.dn.48hrs_dn, pi3k_akt_mtor_signaling, and, in the case of *PA2G4P4*, enrichment of genes in pathways included MYC targets, mTORC1 signaling, genes connected with unfolded protein response, DNA repair, G2M checkpoint, E2F targets, oxidative phosphorylation, and with genes changed after ablation of Rb gene (RB_p107_dn.v1_dn), in the patients with higher expression of *PA2G4P4*. Li et al. described the hypoxia, incistric radiosensitivity, repopulation, and differentiation as the radioresistance parameters for head and neck squamous cell carcinoma. After induction of DNA damage, the cellular checkpoints are activated and lead to cell cycle arrest with the DNA repair or removal of the affected cells [86]. Regulation of mitotic spindle formation, correction of misattached kinetochore-microtubules, spindle checkpoint signaling, and chromatid separation process prevents negative influence of internal and external [69]. Separation of sister chromatids is controlled by the spindle checkpoint, and failure in this causes aneuploidy/polyploidy [87]. Chromosomal instability (CIN) arises from defects in the mitotic checkpoint, altered kinetochore-microtubule dynamics, centrosome amplification, and exposure to ionizing radiation. CIN can influence cancer progression in three distinct ways: it can promote tumor development, suppress tumorigenesis, or have no significant effect, depending on the genetic and cellular context [88]. In the presented work, we observed that higher levels of *PA2G4P4* is associated with higher levels of silent and nonsilent mutation rates, and for higher levels of *ANXA2P2* with lower aneuploidy score and homologous recombination defects. CIN-induced cell death, resulting from the loss of essential chromosomes, represents a potential therapeutic strategy for enhancing radiotherapy efficacy through the addition of CIN-inducing agents [88]. The overcoming of the threshold of CIN could be beneficial. Lee et al. ranked 62 different anticancer drugs for its potential to be used as modifiers of CIN using the model based on human artificial chromosome (HAC). They indicated that paclitaxel, gemcitabine, dactylolide, LMP400, talazoparib, olaparib, peloruside A, GW843682, VX-680, and cisplatin had the most affecting loss of HAC at a high frequency and could be used to lead the cells to the CIN phenotype in the context of radiotherapy [89]. It should be noted that genomic instability is characteristic and frequently occurring in HNSCC. However, this remains speculative, and further in vitro studies are necessary to establish a direct mechanistic link between *ANXA2P2*, *PA2G4P4*, and radiotherapy response [90].

It should be noted that based on our pathway analysis, we can find the upregulation of genes in the group of patients with higher levels of *ANXA2P2* and *PA2G4P4* which are described as radiation resistance pathways [91], and could be useful for target therapy in HNSCC [92]. We suspect that patients with better response to radiotherapy, which as we identified were associated with lower levels of *ANXA2P2* and *PA2G4P4*, the response to radiotherapy could be connected with changes in those pathways. However, further in vitro studies are necessary to clarify the mechanistic links between these pseudogenes and radiotherapy response.

Finally, we investigated the association between high and low *ANXA2P2* and *PA2G4P4* expression levels and the immune profile of HNSCC, integrating our findings with the immune profiling data reported by Thorsson et al. [49]. We observed that patients with lower expression of *PA2G4P4* displayed a higher fraction of CD4 memory-resisting T cells, while patients with lower expression displayed better survival rates. Low expression of *PA2G4P4* was associated with the presence of regulatory T cells (Tregs). Tregs are believed to be pro-cancer [93,94]. However, Liang et al. observed that HNSCC patients with higher levels of Tregs cells have better OS and DFS [95]. Lyu et al. indicated that the pseudogene and parental gene, *HLA-DPB2/HLA-DPB1*, axis was strongly associated with high immune infiltration of CD8+ T cells, CD4+ T cells, Tfh, Th1, and NK cells, and with high expression of majority biomarkers of monocytes, NK cell, T cell, CD8+ T cell, and Th1 in breast cancer. Moreover, HLA-DPB2 and its parental gene *HLA-DPB1* are correlated with better patients’ prognosis and positively correlated with the expression of programmed cell death factor 1 (*PD-1),* its ligand *(PDL-1)* and *CTLA-4* [95,96]. Kim et al. showed that B cells are activated by irradiation and PD-1 blockade, and improve patients’ survival of HPV(+) HNSCC [97]. It was shown that a high fraction of activated (CD86+), antigen-presenting (CD86+/CD21−) and memory B cells (IgD−/CD27+) as well as high infiltration of T follicular helper cells and plasma cells were found in the HNSCC tumors [97]. We observed that in patients with low *PA2G4P4* expression, the naïve B cells were upregulated and memory B cells were downregulated. Previous reports indicated that the role of tumor infiltrating lymphocytes (TILs) is complex and might be affected by a number of factors with pro- or anticancer activity [98]. We also observed M1 type macrophages in patients with lower expression of *PA2G4P4* pseudogene. The postulated polarized macrophage presence in specific tumor regions is essential for OSCC [99] and only M2-like marker CD163+ predicts poor prognosis in HNSCC [100]. The above findings indicate complexity of HNSCC immunity and suggest that pseudogenes may display either direct or indirect effect on HNSCC microenvironment. There has been increasing evidence of the clinical importance of stromal and immune cells in the tumor microenvironment [101]. Those cells are involved in tumor growth, invasion, and metastasis [102,103]. We found that high expression of *ANXA2P2* and *PA2G4P4* correlated with lower stromal scores. High expression of *PA2G4P4* correlated negatively with the immune score. There is no data related to the *PA2G4P4* in cancer immunology.

Our study is limited by the disparity in sample sizes between the control and tumor groups, which may influence the robustness of the findings. To address this limitation, future research should focus on validating these findings using larger, well-balanced cohorts of HNSCC and normal tissue samples. Additionally, the inclusion of matched adjacent normal tissues from the same patients could help minimize variability and strengthen the reliability of the conclusions. Furthermore, a significant challenge in validating our findings is the lack of suitable data on *ANXA2P2* and *PA2G4P4* in publicly available datasets, such as the Gene Expression Omnibus, which could be used for independent in silico validation and comparison with our results derived from the TCGA. While this limitation prevents us from conducting direct validation within external datasets, we acknowledge this gap and emphasize the need for further investigations using independent datasets, either computationally or experimentally through in vitro and in vivo models.

Despite these limitations, this report represents an important step in characterizing the potential oncogenic roles of *ANXA2P2* and *PA2G4P4* in HNSCC. As the first study to explore the involvement of these pseudogenes in this malignancy, our findings serve as a basis for future research.

## 5. Conclusions

Based on the presented results, we hypothesize that the identified pseudogenes *ANXA2P2* and *PA2G4P4* may have potential significance in the pathogenesis of HNSCC. However, definitive evidence of their involvement must be confirmed through in vitro or in vivo models. Based on the results we conclude (1) *ANXA2P2* and *PA2G4P4* pseudogenes contribute to HNSCC pathogenesis. Both pseudogenes were significantly overexpressed in HNSCC tumor samples compared to normal tissues. Their expression levels were associated with tumor localization and HPV status, indicating their potential role in tumor development. (2) Potential oncogenic role of *ANXA2P2* and *PA2G4P4*. High expression of these pseudogenes correlated with the activation of oncogenic signaling pathways. Moreover, the expression levels were associated with TCGA tumor subtypes, proliferation rates, and wound healing ability, further supporting their classification as oncogenes. (3) Patients with low expression of *ANXA2P2* and *PA2G4P4* had significantly longer overall survival. Here, we noticed lower expression levels were also associated with better response to radiotherapy, suggesting their potential predictive value for treatment outcomes. (4) The expression of these pseudogenes was correlated with distinct immune profiles and highlighted their possible role in tumor–immune interactions. In summary, while our findings suggest their potential as biomarkers, further studies are required to fully elucidate their molecular mechanisms and clinical utility in cancer pathogenesis.

## Figures and Tables

**Table 1:** Expression levels of *ANXA2P2* and *PA2G4P4* pseudogenes associated with clinicopathological parameters analyzed in all localizations of HNSCC; *t*-test or Mann–Whitney U test; *p* < 0.05 considered significant.

Pseudogene		ANXA2P2		PA2G4P4	
Parameter	Group	Mean ± SEM	*p*-Value	Mean ± SEM	*p*-Value
Age	>60	2.466 ± 0.0394	0.3555	3.018 ± 0.0290	0.1259
	≤60	2.397 ± 0.0458		2.966 ± 0.0266	
Gender	Female	2.555 ± 0.0508	0.0079	3.039 ± 0.0353	0.0618
	Male	2.388 ± 0.0364		2.972 ± 0.0236	
Alcohol	Positive	2.405 ± 0.0386	0.2450	2.968 ± 0.0245	0.1680
	Negative	2.473 ± 0.0496		3.037 ± 0.0328	
Smoking	No/Ex	2.441 ± 0.0352	0.6006	2.990 ± 0.0241	0.7798
	Yes	2.391 ± 0.0571		3.000 ± 0.0350	
Cancer Stage	I + II	2.473 ± 0.0570	0.8922	3.014 ± 0.0447	0.9321
	III + IV	2.479 ± 0.0378		2.998 ± 0.0245	
T Stage	T1 + T2	2.393 ± 0.0458	0.0427	2.965 ± 0.0350	0.7083
	T3 + T4	2.514 ± 0.0432		2.989 ± 0.0251	
N Stage	N0	2.566 ± 0.0442	0.0314	2.913 ± 0.0334	0.0226
	N1+N2+N3	2.395 ± 0.0475		3.019 ± 0.0265	
Grade	G1 + G2	2.504 ± 0.0316	0.0033	2.995 ± 0.0232	0.8218
	G3 + G4	2.267 ± 0.0734		2.985 ± 0.0424	
Perineural invasion	Positive	2.573 ± 0.0448	0.0116	3.025 ± 0.0341	0.2572
	Negative	2.373 ± 0.0548		2.984 ± 0.0354	
Lymph Node Neck Dissection	Positive	2.455 ± 0.0336	0.1344	3.006 ± 0.0223	0.0698
	Negative	2.336 ± 0.0690		2.927 ± 0.0410	
Lympho- vascular Invasion	Positive	2.379 ± 0.0728	0.0921	3.046 ± 0.0426	0.4558
	Negative	2.532 ± 0.0410		2.987 ± 0.0322	
HPV status	Positive	1.739 ± 0.1558	<0.0001	2.912 ± 0.0634	0.6705
	Negative	2.447 ± 0.0740		2.950 ± 0.0554	

## Data Availability

The datasets used and/or analyzed during the current study are available from the corresponding author on reasonable request. Raw data are available on the XenaBrowser, Ualcan, cBioportal, and ESTIMATE databases.

## Supplementary Materials

The following supporting information can be downloaded at https://www.mdpi.com/article/10.3390/biomedicines14010200/s1, Supplementary materials are the integral part of this work. Supplementary Figure S1. Preselection of *ANXA2P2* and *PA2G4P4* pseudogenes based on UALCAN and ENCORI databases: (A) expression levels in healthy and cancer tissue; (B) association of pseudogene expression with patient survival; Graphs downloaded from UALCAN and ENCORI databases, modified; ns—not significant, *** *p* < 0.001, ** *p* < 0.01, and * *p* < 0.05 considered as significant; FDR—false discovery rate; LogRank (Mantel–Cox) test; low and high subgroups of patients divided based on mean expression; *p* < 0.05 considered significant; HR—hazard ratio; 95% CI—confidence interval; coefficient β (Coef β). Supplementary Figure S2. Characteristics of parental genes of *ANXA2P* and *PA2G4P4* pseudogenes: (A) expressions levels of *ANXA2* and *PA2G4* in normal and cancer samples based on the UALCAN database; (B) correlation of parental gene and its pseudogene, *ANXA2* and *ANXA2P2*, *PA2G4* and *PA2G4P4* as well as between two parental genes, *ANXA2* and *PA2G4*, in normal and cancer samples using the GEPIA2 database; (C) expression levels of parental genes depending on the HPV status; HPV(-) and HPV(+) group of patients based on UALCAN database; (D) OS and DFS for parental genes of *ANXA2P2* and *PA2G4P4* based on the GEPIA2 database; p < 0.05 considered significant; HR—hazard ratio; 95% CI—confidence interval. Supplementary Table S1. Spearman R values—correlation matrix between *ANXA2P* and *PA2G4P4* expression levels. Supplementary Table S2. Association of *ANXA2P* and *PA2G4P4* expression levels and classification to the atypical, basal, classical and mesenchymal tumor subtype based on the TCGA characteristics, proliferation ratio, wound healing ability and intratumor heterogeneity; *p* < 0.05 considered as significant. Supplementary Table S3. Genes are negatively or positively correlated with *ANXA2P* and *PA2G4P4*, and classified into cellular processes and pathways based on REACTOME analysis. Supplementary Table S4. List of processes and genes enriched between groups of patients with high expression of *ANXA2P* and *PA2G4P4* based on GSEA. Supplementary Table S5. Association of *ANXA2P* and *PA2G4P4* with the response to ionizing radiation. Supplementary Table S6. Association of the expression levels of *ANXA2P* and *PA2G4P4* pseudogenes with the immune profile of HNSCC patients. Supplementary Table S7. Immunological profile of HNSCC patients associated with the expression level of *ANXA2P* and *PA2G4P4* pseudogenes.

## Abbreviations

CI	Confidence Interval
DFS	Disease Free Survival
ESTIMATE	Estimation of STromal and Immune cells in MAlignant Tumor tissues using Expression data
FDR	False Discovery Rate
GSEA	Gene Set Enrichment Analysis
HNSCC	Head and Neck Squamous Cell Carcinoma
HPV	Human papillomavirus
MHC	Major Histocompatibility Complex
NES	Normalized Enrichment Score
OS	Overall Survival
ROC	Receiving-operating characteristic
TCGA	The Cancer Genome Atlas
